# Weight at Birth and Subsequent Fecundability: A Prospective Cohort Study

**DOI:** 10.1371/journal.pone.0095257

**Published:** 2014-04-15

**Authors:** Cathrine Wildenschild, Anders H. Riis, Vera Ehrenstein, Berit L. Heitmann, Elizabeth E. Hatch, Lauren A. Wise, Kenneth J. Rothman, Henrik T. Sørensen, Ellen M. Mikkelsen

**Affiliations:** 1 Department of Clinical Epidemiology, Aarhus University Hospital, Aarhus, Denmark; 2 Institute of Preventive Medicine, Bispebjerg and Frederiksberg Hospital, Copenhagen University and National Institute of Public Health, University of Southern Denmark, Copenhagen, Denmark; 3 The Boden Institute of Obesity, Nutrition Exercise & Eating Disorders, University of Sydney, Sydney, New South Wales, Australia; 4 Department of Epidemiology, Boston University School of Public Health, Boston, Massachusetts, United States of America; 5 Slone Epidemiology Center, Boston University, Boston, Massachusetts, United States of America; 6 RTI Health Solutions, Research Triangle Park, North Carolina, United States of America; University of Nevada School of Medicine, United States of America

## Abstract

**Objective:**

To examine the association between a woman's birth weight and her subsequent fecundability.

**Method:**

In this prospective cohort study, we included 2,773 Danish pregnancy planners enrolled in the internet-based cohort study “Snart-Gravid”, conducted during 2007–2012. Participants were 18–40 years old at study entry, attempting to conceive, and were not receiving fertility treatment. Data on weight at birth were obtained from the Danish Medical Birth Registry and categorized as <2,500 grams, 2,500–2,999 grams, 3,000–3,999 grams, and ≥4,000 grams. In additional analyses, birth weight was categorized according to z-scores for each gestational week at birth. Time-to-pregnancy measured in cycles was used to compute fecundability ratios (FR) and 95% confidence intervals (CI), using a proportional probabilities regression model.

**Results:**

Relative to women with a birth weight of 3,000–3,999 grams, FRs adjusted for gestational age, year of birth, and maternal socio-demographic and medical factors were 0.99 (95% CI: 0.73;1.34), 0.99 (95% CI: 0.87;1.12), and 1.08 (95% CI: 0.94;1.24) for birth weight <2,500 grams, 2,500–2,999 grams, and ≥4,000 grams, respectively. Estimates remained unchanged after further adjustment for markers of the participant's mother's fecundability. We obtained similar results when we restricted to women who were born at term, and to women who had attempted to conceive for a maximum of 6 cycles before study entry. Results remained similar when we estimated FRs according to z-scores of birth weight.

**Conclusion:**

Our results indicate that birth weight appears not to be an important determinant of fecundability.

## Background

Several studies have shown that individuals with a low weight at birth are at increased risk of developing morbidities in adulthood, possibly due to physiologic, metabolic, and hormonal changes during fetal life associated with insufficient growth [1]–[4]. Being born small for gestational age (SGA) is associated with earlier onset of puberty and menarche [5]–[8], and with abnormalities in ovarian development and functioning among adolescent girls, such as reduced uterine and ovarian size, lower ovulation rate and anovulation, and ovarian hyporesponsiveness to follicle stimulating hormone [9]–[12]. It is uncertain whether potentially compromised ovarian development and function in early life persist into adulthood and have long-term effects on reproduction.

A reduced probability of giving birth has been reported among women born before 32 full weeks [13]–[15] and among women born with a very low birth weight (<1500 grams) [13], [15], [16]. The few studies that have examined the association between birth weight and later ability to conceive had conflicting findings [17], [18]. In the Danish National Birth Cohort, Nohr et al. reported an odds ratio for a time-to-pregnancy (TTP) greater than 12 months (indicative of infertility) of 1.2 (95% CI: 1.0;1.5) among women born at term with a weight ≤2,500 grams, and 1.8 (95% CI: 1.1;3.1) among women born preterm with a weight ≤1,500 grams, compared with women born at term with a weight of 3,001–4,000 grams [18]. In contrast, Meas et al. reported no increase in TTP among French women born SGA [17]. Both studies were restricted to women who became pregnant and therefore assessed TTP conditional on the achieved pregnancy, using retrospectively collected TTP data. To our knowledge, no study has examined fecundability (i.e., the cycle-specific probability of conception) according to weight at birth.

Whether the association between weight at birth and subsequent health is attributable to direct effects of insufficient fetal growth or to underlying shared mechanisms, i.e., intergenerational factors with a potential influence on fetal growth and adult health, has been the subject of debate [19], [20]. Familial clustering has been reported for extremes of birth weight [21], preterm birth [22]–[26], spontaneous abortion [27]–[29], and family size [30]–[32]. Little is known, however, about intergenerational patterns in fecundability. Reproductive characteristics of a woman's mother, such as number of children, difficulty conceiving, or history of spontaneous abortion may be proxy markers of the mother's fecundability, and in turn may affect fecundability of the woman. Several studies have found that mother's parity [13], [15], [33], mother's history of spontaneous abortion [34], [35], and mother's history of infertility [36]–[38] were associated with low birth weight in her offspring. These findings imply that maternal fecundability could confound the putative association between daughter's birth weight and her fecundability. This potential confounding was not controlled in previous studies.

We examined the association between weight at birth and subsequent fecundability of women participating in a prospective cohort study of TTP, while controlling for potential confounding by reproductive characteristics of the women's mothers.

## Subjects and Methods

### Study population

In this study, we used data from the “Snart-Gravid” (“Soon Pregnant”) study, which is a Danish internet-based prospective cohort study of pregnancy planners, designed to examine the influence of lifestyle and behavioral factors on fecundability. The study design and data collection have been described in detail elsewhere [39]. Briefly, participants were recruited and followed via the internet during 2007–2012. Eligible women were aged 18–40 years, in a stable relationship with a male partner, attempting to conceive, and not receiving fertility treatment. After giving informed consent, participants provided their Civil Personal Registration (CPR) number, a unique personal identifier assigned to all Danish citizens at birth. The CPR number permits unambiguous identification and linkage of persons in Danish administrative and medical registries [40]. At enrollment, participants completed a baseline questionnaire with items on demographics, lifestyle and behaviors, and medical and reproductive history, including months of trying to conceive. Participants subsequently completed bimonthly follow-up questionnaires until they reported pregnancy, discontinuation of pregnancy attempts, beginning of fertility treatment, or had been followed for 12 months (end of study observation), whichever came first. Follow-up questionnaires elicited information on changes in relevant exposures and whether pregnancy had occurred.

By August 2012, 6,033 women had enrolled in the study by responding to the baseline questionnaire. We excluded 579 women who did not complete a follow-up questionnaire, 113 repeated entries, 294 women with implausible or missing information on date of last menstrual period, 538 women who had attempted to conceive for more than 11 cycles at enrollment, and 226 women who had been adopted, born after a non-singleton gestation, or had missing data on multiplicity of gestation. In order to obtain uniformly recorded data on gestational age at birth from the Danish Medical Birth Registry (DMBR), we also excluded 1,510 women who were born before January 1, 1978. The remaining 2,773 women were included in the analyses.

### Measures of weight at birth

We obtained data on the participants' weight at birth from the DMBR. This registry records over 99% of births in Denmark, reported prospectively by midwives attending the birth [41]. Data on birth weight were registered in categories of 250 grams in 1978, in categories of 10 grams during 1979–1990, and in exact grams after 1990 [42]. We categorized birth weight as <2,500, 2,500–2,999, 3,000–3,999, and ≥4,000 grams, and used 3,000–3,999 grams as the reference category. In additional analyses, we estimated z-scores for birth weight by each completed gestational week as (participant's birth weight – mean of birth weights for the gestational week of birth)/(the standard deviation of the mean of birth weights for the gestational week of birth) [43]. Estimation of mean birth weight and standard deviation in each gestational week was based on the birth weight distribution of Danish girls in the period 1978–1992 (i.e., the period of the participants' births), as registered in the DMBR. The z-scores were then grouped into 6 categories of ≤-2, -2-≤-1, -1-≤0, 0-≤1, 1-≤2, and >2, with 0-≤1 as the reference category.

### Measures of time-to-pregnancy (TTP)

The event of interest was participants' report of any pregnancy regardless of outcome. More than 96% of participants used a home pregnancy test to confirm conception [44]. At each follow-up, participants reported the date of their last menstrual period (LMP), whether they were currently pregnant, and occurrence since the previous follow-up of spontaneous abortion, therapeutic abortion, or ectopic pregnancy. Total number of menstrual cycles at risk of pregnancy (i.e., TTP) was calculated as (days of attempt time at study entry/usual cycle length)+(((LMP date from most recent follow-up questionnaire – date of study entry)/usual cycle length)+1). Participants could contribute information until their 12^th^ cycle of attempted pregnancy to the analysis. Observed cycles at risk of pregnancy were defined as cycles contributed after study enrollment and were left-truncated. Thus, if a woman had already attempted to conceive for 8 cycles when she entered the study, she could contribute up to 4 more cycles after enrollment into the study, with her observed cycles starting at cycle 9 (delayed entry). The follow-up of women who started fertility treatment during follow-up was censored at the cycle in which they started the treatment.

### Covariates

We obtained data on participants' gestational age at birth from the DMBR. Data on gestational age were based on the date of the pregnant woman's last menstrual period, corrected by ultrasound examination if performed, and registered in full weeks. Gestational ages of the participants were 28–44 completed weeks. We defined preterm to be a gestational age <37 weeks; full term to be 37–41 weeks; and post-term to be ≥42 weeks. From the DMBR, we also obtained information on participants' mothers' lifetime parity and participants' birth order by using the CPR number to identify mothers and siblings. Siblings born before establishment of the DMBR in 1973 were identified by the mothers' self-reported parity, which was also registered in the DMBR and has high validity [45]. Data on mothers' lifetime parity were divided into categories 1 (study participant was an only child), 2–3 children, and ≥4 children (reference category). Participants' birth order was categorized as first-born, second-born, or greater than second-born (reference category). Data on participants' mothers' history of difficulty conceiving (yes/no) and history of spontaneous abortion (yes/no) were reported in the baseline questionnaire, and we defined participants' mothers without such history as the reference category. Reference categories were defined on the assumption that they represented mothers with normal fecundability.

From the DMBR we obtained data on mother's age and marital status at the time the participant was born. From the Danish National Registry of Patients (DNRP), which includes data on all admissions to Danish non-psychiatric hospitals since 1977, we obtained data on hospital diagnoses of hypertension or pre-eclampsia during the mother's pregnancy with the participant. These diagnoses were coded according to ICD-8 during the period of interest. We used ICD-8 codes 400–404 and 637.00 (essential and gestational hypertension) and 637.03, 637.04, 637.09, 637.19, and 637.99 (pre-eclampsia, eclampsia, and toxemia). Prevalence of hospital admission due to maternal diabetes was below 1%, therefore maternal diabetes as measured by hospitalization was not a strong confounder in our analysis.

From the baseline questionnaire we obtained data on participants' own reproductive history, including age at menarche, cycle regularity, gravidity, parity, and history of unsuccessful pregnancy attempts ≥12 months. At baseline, participants also reported their weight (in kilograms) and height (in centimeters) and we calculated their body mass index (BMI) as (weight (kilograms)/height squared (m^2^)). Further, data on participants' age, number of cycles of pregnancy attempt at study entry, intercourse frequency, mother's and father's educational level, and mother's smoking during pregnancy were reported in the baseline questionnaire.

### Ethics statement

The “Snart-Gravid” study was approved by the Danish Data Protection Board (journal no. 2013-41-1922) and the Institutional Review Board at Boston University. Consent was obtained from the participants before completion of the first questionnaire. Data from the DMBR and the DNRP were retrieved from Statens Serum Institut (http://www.ssi.dk/Sundhedsdataotit.aspx). Data from the ”Snart-Gravid” study are hosted by the Department of Clinical Epidemiology, Aarhus University Hospital; as this study is still in progress, access to the data is not yet freely available. All data were anonymized after retrieval and no CPR numbers were included in the dataset that was the basis of our analyses.

### Missing values

The proportion of missing values for the variables birth weight, birth order, mother's lifetime parity, mother's age at delivery, mother's marital status, and mother's smoking during pregnancy ranged from 4.8% to 8.4%. For 17.2% of the participants, values were missing on gestational age at birth, which was partly attributable to procedural changes instituted in 1978 in reporting this variable to the DMBR [46]. For 17.2% and 20.4% of participants, there were missing observations on mother's history of difficulty conceiving and mother's history of spontaneous abortion, respectively, most likely due to participants not knowing this information. For 30.4% and 35.0% of participants, there were missing observations on mother's and father's educational level, respectively. These missing data resulted from random assignment of half of the early study participants to a short-form baseline questionnaire that did not include questions on parental educational level.

On the assumption that data were missing at random, we imputed missing values using multiple imputation by chained equations (MICE program in Stata version 12.0). We included 36 variables in the imputation, including all variables used in the substantive analyses, and imputed five data sets. Distributions of continuous variables were examined by histograms and box plots. Variables that diverged from the normal distribution were transformed to the log-scale before imputation.

### Data analysis

We calculated Kaplan-Meier estimates to assess the cumulative probability of conception within 3, 6, and 12 menstrual cycles, accounting for delayed entry using left-truncation, and losses to follow-up and other reasons for censoring (e.g., no longer trying to conceive or initiation of fertility treatment). We described the distribution of participants' characteristics (for women lost to follow-up, women who completed the study, and for all of the 2,773 women in the study cohort) according to weight at birth. Using a proportional probabilities model, we then estimated fecundability ratios (FR) and 95% confidence intervals (CI) for categories of birth weight (<2,500, 2,500–2,999, and ≥4,000 grams, with 3,000–3,999 grams as the reference category), using TTP measured in cycles. The FR of any two groups was calculated as the ratio of their cycle-specific probabilities [47]. Participants contributed cycles at risk from entry into the study until report of pregnancy, receipt of fertility treatment, discontinuation of pregnancy attempt, loss to follow-up, or end of observation (maximum 12 cycles). Distinct intercept parameters were included for each of the 12 cycles of follow-up, to allow for decline in the baseline conception rate over follow-up time.

We examined potential interaction between weight and gestational age at birth by including product terms for gestational age as a continuous variable in the regression model, and found no evidence of interaction. Adjustments were made in three steps: first, we adjusted for year of birth and gestational age as a continuous variable with values 28–44 weeks only (model 1); second, we included parental socio-demographic and medical characteristics (mother's age, mother's marital status, mother's and father's educational level, mother's smoking during pregnancy, and mother's history of hypertension and pre-eclampsia) (model 2); and third, we included markers of the participant's mother's fecundability in the regression model (mother's lifetime parity, participant's birth order, mother's history of difficulty conceiving, and mother's history of spontaneous abortion) (model 3). Variables included in the three models were chosen a priori because they have previously been associated with offspring weight at birth [13], [15], [33]–[38], [48]–[51], and may influence the daughter's fecundability [13], [15], [27]–[32], [52], [53]. Not much is known about the potential influence of maternal reproductive health on the fecundability of daughters. Based on evidence of familial clustering of other reproductive health outcomes [21]–[32], it is plausible that proxy markers of the mother's fecundability, e.g., mother's history of difficulty conceiving, might be causally associated with daughters' fecundability. On this basis, we investigated the potential confounding effect of maternal socio-demographic, medical and reproductive characteristics. We repeated the analyses restricted to women born at term, i.e., at 37–41 weeks of gestation, to restrict the influence of gestational age at birth. To evaluate sensitivity of the study result to inclusion of women who had tried to conceive for up to 11 cycles at study entry, we repeated the analyses restricted to women with only up to 6 cycles of attempt time. Previous reports indicate that accelerated weight gain in infancy, which is often exhibited by infants with a low birth weight, is associated with overweight or obesity later in life [54], [55], and obesity has been linked with reduced fecundability [56]. Thus, we also considered the potential mediating influence of pre-pregnancy BMI on an association between weight at birth and fecundability.

In addition to considering gestational age at birth by adjustment and restriction to term births, we also examined the association between weight at birth and fecundability by z-scores of birth weight, to compare infants of differing relative weights by using weight estimates that were adjusted for gestational age at birth [43]. We estimated fecundability ratios by categories of z-score (≤-2, -2-≤-1, -1-≤0, 1-≤2, and >2, with 0-≤1 as the reference category), using the same proportional probabilities regression model as in the initial analyses.

Analyses were performed using Stata version 12.0 (StataCorp., TX, USA) and SAS version 9.2 (Cary, NC, USA).

## Results

Among the 2,773 women included in our analyses, 245 (8.8%) were lost to follow-up. Women lost to follow-up contributed cycles at risk for as many cycles as they were observed in the study, and were censored at the time of non-response. Among women lost to follow-up, mean birth weight overall was 3,281 grams (95% CI: 3,209;3,353 grams), which was slightly lower than among women with complete follow-up. The distribution of gestational age at birth among women lost to follow-up was similar to that for women who completed the study (data not shown). Women with low birth weight that were lost to follow-up were more likely to have a mother who was divorced or widowed, and had a lifetime parity of ≥4 children, more likely to have a high birth order and irregular cycles, and more had only attempted to become pregnant for 0–1 cycles at study entry, compared with women with low birth weight who completed the study (data not shown).

Mean birth weight overall among the 2,773 women in the study cohort was 3,315 grams (95% CI: 3,295;3,334 grams), and mean birth weight among those born at term was 3,326 grams (95% CI: 3,307;3,345 grams). There were 2,432 (87.7%) participants who had been born at term, 102 (3.7%) who had been born preterm, and 239 (8.6%) who had been born post-term.

Kaplan-Meier estimates for the cumulative probability of conception among the 2,773 participants were 47% within 3 cycles, 67% within 6 cycles, and 83% within 12 cycles. Characteristics of participants according to their weight at birth are presented in Table 1. Participants with a birth weight <2,500 grams were more likely to have been exposed to maternal smoking in pregnancy, have a mother who had hypertension or pre-eclampsia during pregnancy with the participant, have a mother with a history of difficulty conceiving or spontaneous abortion, have a mother with a lifetime parity of at least 4 children, and to be first-born. They were also more likely to be obese (BMI≥30), to have a history of unsuccessful pregnancy attempts ≥12 months, longer pregnancy attempt time at study entry, and intercourse ≥4 times a week, compared with participants with a birth weight of 3,000–3,999 grams.

**Table 1:** Characteristics of 2,773 women according to categories of birth weight, “Snart-Gravid” study, Denmark, 2007–2012.

	Birth weight, grams			
Characteristic	<2,500	2,500–2,999	3,000–3,999	≥4,000
No. of women	119	488	1,866	300
Age, years (mean)	26.1	26.4	26.5	26.5
Born at term (%)	54.6	89.8	90.5	80.3
Mother's age at time of delivery (median)	25	25	26	26
Mother's marital status (%):				
Married	61.3	62.1	65.1	71.7
Unmarried	31.1	34.4	31.2	24.7
Divorced/widowed	7.6	3.5	3.7	3.7
Mother's education, less than Upper Secondary School (%)	69.8	60.9	57.2	59.0
Father's education, less than Upper Secondary School (%)	74.0	64.6	67.3	71.7
Mother smoked during pregnancy (%)	57.1	51.8	31.4	22.0
Mother had hypertension (%)*	3.4	0.4	0.8	1.0
Mother had pre-eclampsia (%)*	7.6	3.3	1.6	2.7
Mother had difficulty conceiving (%)	19.3	18.9	13.3	15.0
Mother had spontaneous abortion (%)	42.0	28.9	24.5	18.3
Mother's lifetime parity (%):				
1	10.9	12.1	9.4	6.3
2–3	68.9	74.6	76.9	76.0
≥4	20.2	13.3	13.7	17.7
Birth order of participant (%):				
First-born	54.6	56.4	45.2	32.0
Second-born	27.7	29.7	37.1	47.0
>Second-born	17.7	13.9	17.7	21.0
Age at menarche, years (mean)	12.6	12.7	12.9	12.9
Irregular cycles (%)	26.1	25.0	28.7	27.7
Gravidity ≥1 (%)	32.8	37.3	33.1	33.0
Parity ≥1 (%)	21.0	21.7	20.0	20.3
History of unsuccessful pregnancy attempts ≥12 months (%)	16.8	11.9	7.8	6.3
Pre-pregnancy BMI, kg/m^2^ (%):				
<18.5	5.9	5.9	4.0	3.0
18.5–24.9	53.8	60.5	64.6	62.0
25.0–29.9	21.9	18.0	20.3	22.7
≥30	18.5	15.6	11.1	12.3
No. of cycles of pregnancy attempt at study entry (%):				
0–1	41.2	48.6	47.7	46.7
2–3	23.5	22.8	21.9	27.0
4–6	21.0	16.4	17.3	17.7
7–11	14.3	12.3	13.1	8.7
Intercourse frequency ≥4 times/week (%)	26.1	22.8	21.1	23.0

*Mother diagnosed with hypertension or pre-eclampsia during pregnancy with the participant.

Crude and adjusted FRs according to weight at birth are presented in Table 2. After adjustment for all covariates except BMI and measures of maternal fecundability (model 2), FRs for birth weight categories <2,500 grams, 2,500–2,999 grams and ≥4,000 grams, compared with the reference category, were 0.99 (95% CI: 0.73;1.34), 0.99 (95% CI: 0.87;1.12), and 1.08 (95% CI: 0.94;1.24), respectively. When we added markers of maternal fecundability to the regression analysis (mother's lifetime parity, participant's birth order, mother's history of difficulty conceiving, and mother's history of spontaneous abortion) (model 3), we obtained almost identical results; FRs were 0.98 (95% CI: 0.72;1.32), 0.99 (95% CI: 0.87;1.13), and 1.07 (95% CI: 0.93;1.24) for birth weights <2,500 grams, 2,500–2,999 grams, and ≥4,000 grams, respectively.

**Table 2:** Fecundability by categories of birth weight.

					Unadjusted model		Adjusted model^1^		Adjusted model^2^		Adjusted model^3^	
	Birth weight, grams	No. of women	No. of cycles	No. Of pregnancies	FR	95% CI	FR	95% CI	FR	95% CI	FR	95% CI
All women,												
N = 2,773	<2,500	119	504	66	0.89	0.71;1.12	1.01	0.75;1.36	0.99	0.73;1.34	0.98	0.72;1.32
	2,500–2,999	488	1,979	314	0.97	0.86;1.09	1.00	0.88;1.13	0.99	0.87;1.12	0.99	0.87;1.13
	3,000–3,999	1,866	7,461	1,176	1.00	Reference	1.00	Reference	1.00	Reference	1.00	Reference
	≥4,000	300	1,131	201	1.10	0.96;1.26	1.07	0.94;1.23	1.08	0.94;1.24	1.07	0.93;1.24
Born at term,												
N = 2,432	<2,500	65	230	36	0.98	0.69;1.38	1.01	0.70;1.46	1.01	0.69;1.46	1.00	0.69;1.45
	2,500–2,999	452	1,786	277	0.96	0.84;1.09	0.97	0.85;1.11	0.96	0.84;1.10	0.97	0.84;1.12
	3,000–3,999	1,814	6,782	1,069	1.00	Reference	1.00	Reference	1.00	Reference	1.00	Reference
	≥4,000	279	947	166	1.11	0.95;1.29	1.10	0.94;1.28	1.09	0.93;1.27	1.08	0.93;1.26

**Model^1^**: Adjusted for participant's gestational age and year of birth.

**Model^2^**: Model 1 + mother's age, mother's marital status, mother's and father's educational level, mother's smoking during pregnancy, mother's hypertension, and mother's pre-eclampsia during pregnancy with the participant.

**Model^3^**: Model 2 + mother's lifetime parity, participant's birth order, mother's history of difficulty conceiving, and mother's history of spontaneous abortion.

Table 2 shows that results changed little after restricting the analysis to women born at term. Relative to women with a birth weight of 3,000–3,999 grams, FRs in the fully adjusted model were 1.00 (95% CI: 0.69;1.45), 0.97 (95% CI: 0.84;1.12), and 1.08 (95% CI: 0.93;1.26) for women with a birth weight <2,500 grams, 2,500–2,999 grams, and ≥4,000 grams, respectively. Repeating these analyses among women with up to 6 cycles of pregnancy attempt at study entry yielded similar results (data not shown). Results were also consistent when we controlled for pre-pregnancy BMI via stratification or adjustment (data not shown). As shown in Table 3, when we examined the association between weight at birth and fecundability using z-scores, we obtained results similar to those based on absolute measures of weight at birth, i.e., FRs suggested little association.

**Table 3:** Fecundability by z-scores of birthweight for gestational age.

					Unadjusted model		Adjusted model^1^		Adjusted model^2^		Adjusted model^3^	
	Z-score of birthweight	No. of women	No. of cycles	No. of pregnancies	FR	95% CI	FR	95% CI	FR	95% CI	FR	95% CI
All women,												
N = 2,773	≤-2	28	99	17	1.23	0.78;1.92	1.19	0.76;1.87	1.17	0.74;1.85	1.17	0.74;1.86
	-2-≤-1	379	1,523	246	1.07	0.92;1.24	1.06	0.91;1.23	1.04	0.89;1.21	1.04	0.89;1.22
	-1-≤0	1,127	4,512	713	1.03	0.92;1.14	1.02	0.92;1.14	1.02	0.91;1.13	1.02	0.91;1.13
	0-≤1	915	3,693	566	1.00	Reference	1.00	Reference	1.00	Reference	1.00	Reference
	1-≤2	298	1,143	199	1.12	0.96;1.30	1.11	0.96;1.29	1.11	0.95;1.29	1.10	0.95;1.28
	>2	26	105	16	0.98	0.62;1.55	0.95	0.60;1.51	0.95	0.59;1.52	0.95	0.59;1.51
Born at term,												
N = 2,432	≤-2	27	96	16	1.17	0.72;1.88	1.15	0.71;1.85	1.14	0.70;1.85	1.13	0.69;1.85
	-2-≤-1	325	1,348	208	0.98	0.84;1.14	0.97	0.83;1.14	0.97	0.83;1.14	0.97	0.82;1.14
	-1-≤0	1,011	4,042	642	0.99	0.88;1.11	0.99	0.88;1.11	0.99	0.88;1.11	0.99	0.88;1.11
	0-≤1	776	3,092	487	1.00	Reference	1.00	Reference	1.00	Reference	1.00	Reference
	1-≤2	268	1,063	180	1.05	0.89;1.23	1.05	0.89;1.23	1.04	0.88;1.22	1.03	0.88;1.21
	>2	25	104	15	0.91	0.57;1.44	0.89	0.56;1.41	0.89	0.56;1.42	0.89	0.56;1.41

**Model^1^**: Adjusted for participant's year of birth.

**Model^2^**: Model 1 + mother's age, mother's marital status, mother's and father's educational level, mother's smoking during pregnancy, mother's hypertension, and mother's pre-eclampsia during pregnancy with the participant.

**Model^3^**: Model 2 + mother's lifetime parity, participant's birth order, mother's history of difficulty conceiving, and mother's history of spontaneous abortion.

## Discussion

In our study of 2,773 pregnancy planners, we found little evidence supporting a relation between weight at birth and fecundability. Results were similar when we restricted the cohort to women born at term, and when we considered relative measures of weight at birth using z-score transformation. Further, we found no indication that markers of maternal fecundability confounded the association between weight at birth and women's own fecundability.

To our knowledge, this is the first prospective study to examine the association between weight at birth and fecundability in a cohort of pregnancy planners. Our data allowed for a more accurate estimate of TTP, based on women with and without successful conceptions, in contrast to data retrospectively obtained from women who were already pregnant. A validation study of retrospective data on TTP, using prospective data as the gold standard, reported a mean difference in TTP of −1.4 months among women with a recall period of 3–20 months [57], suggesting that misclassification of TTP may be present in retrospective studies, even for recent pregnancies. While the “Snart-Gravid” study may appeal more to women who anticipate that their fecundability may be impaired, it is unlikely that participation would be related to weight at birth, as participants had no knowledge that these associations would be investigated when they entered the study. When we restricted our analysis to women with a maximum of 6 cycles of pregnancy attempt time at study entry to assess the potential influence of excluding women who may have had reduced fecundability, our findings were similar. The proportion of women with low birth weight was slightly higher among those lost to follow-up. In addition, among women with low birth weight who were lost to follow-up, more had irregular cycles, and more had only attempted to become pregnant for 0–1 cycles at study entry, compared with women with low birth weight who completed the study. However, differential loss to follow-up is unlikely to have attenuated our findings, as there was little association with fecundability for any category of birth weight.

Data on birth weight were not recorded in a uniform manner in the DMBR during the birth years of the participants in our cohort [42]. The resulting non-differential misclassification of birth weight may have diluted the association if there was one. Nevertheless, by using registry-based data on weight and gestational age at birth, we avoided the possibility of differential misclassification. It is known that preterm birth was underreported to the DMBR during the birth years of our cohort [41]; however, there was little association of low birth weight with fecundability before adjustment for gestational age. Small numbers precluded us from examining the association of fecundability with very low birth weight (<1,500 grams), which has been associated with prolonged TTP and reduced probability of reproducing in similar studies [13], [15], [16], [18]. Therefore, our inability to differentiate birth weights of <1,500 grams from those <2,500 grams may have obscured an association for very low birth weight.

In agreement with our results, a French prospective study of 403 women who had attempted to conceive found nearly no association between being born SGA and later TTP, relative to women whose size at birth was appropriate for gestational age [17]. Similarly, a registry-based prospective study of 148,281 Swedish women found little association between being born SGA and the probability of giving birth, when SGA was defined as 3 standard deviations below the mean weight for the length of gestation [13]. Likewise, a registry-based study of 494,692 Swedish women (including women from the other Swedish study [13]) found little association between being born SGA and the probability of giving birth. This study also reported a hazard ratio for giving birth of 0.95 (95% CI: 0.93; 0.97) among women with a birth weight <2,500 grams [15]. These results appear to support our findings, though we recognize that actual reproduction cannot be equated to fecundability; thus, the Swedish studies do not necessarily convey information on potential differences in the ability to conceive according to weight at birth.

Our findings differ from those of Nohr et al., who conducted a retrospective TTP study of 21,786 Danish women and reported an OR for a TTP of 6–12 months of 1.2 (95% CI: 0.9;1.5) and OR for a TTP >12 months of 1.2 (95% CI: 1.0;1.5) among women born at term with a birth weight ≤2,500 grams, compared with women born at term with a weight of 3,001–4,000 grams [18]. The study by Nohr et al. was conducted in a cohort of pregnant women who reported their weight and gestational age at birth, as well as retrospective data on TTP leading to their ongoing pregnancy. As such, results are not directly comparable with ours. Our data indicated that weight at birth is not meaningfully associated with a reduced fecundability; however, even a weak association would be easier to distinguish from a null association in a larger cohort. We do not know whether the associations observed in the other Danish study were causal or due to shared risk factors that were uncontrolled.

In conclusion, the present study indicates that infant weight at birth does not appear to have a meaningful influence on female fertility in adult life. If correct, this finding implies that even if gonadal development and function are compromised in adolescents with a small size at birth, such anomalies may not persist to influence fecundability in adult women attempting to conceive.

## Acknowledgments

The authors wish to thank Tina Christensen for her support with data collection and media contact, and Thomas Jensen for his assistance with website design.
